# Unicystic ameloblastoma in 3 year old paediatric patient – A rare entity

**DOI:** 10.4317/jced.50793

**Published:** 2013-02-01

**Authors:** Shelly Arora, Priya Kumar, Aadithya B. Urs, Jeyaseelan Augustine

**Affiliations:** 1 Senior Research Associate. Department of Oral and Maxillofacial Pathology, Maulana Azad Institute of Dental Sciences, India; 2 Assistant professor. Department of Oral and Maxillofacial Pathology, Maulana Azad Institute of Dental Sciences, India; 3 Professor. Department of Oral and Maxillofacial Pathology, Maulana Azad Institute of Dental Sciences, India

## Abstract

Unicystic ameloblastoma (UA) is a benign epithelial odontogenic tumor of the jaws that commonly occurs in 2nd and 3rd decade of life. In fact, this entity is rare in children under 12 years of age. It is characterised as a distinct variant of ameloblastoma, exhibiting a less aggressive behaviour and a lower rate of recurrence than solid conventional ameloblastoma. There are very few reported cases of UA occurring in children below five years of age. The purpose of this case report is to describe a case of UA involving the crown of an unerupted maxillary second premolar in a 3 year old girl. The pathogenesis, clinical appearance, radiographic presentation, histological findings and management of the tumour have also been discussed.

## Introduction 

Ameloblastoma is one of the most common benign odontogenic tumours, accounting for approximately 1% of all tumours and cysts of the jaws and 10% of all odontogenic tumors (1,2). Unicystic ameloblastoma (UA), refers to those cystic lesions that show clinical and radiological features of an odontogenic cyst but in histological examination show ameloblastomatous epithelium lining the cyst cavity with or without luminal or mural proliferation (3). UA represents 5-15% of ameloblastoma cases (4) and tends to occur in a younger population as compared to conventional ameloblastoma (5,6). This article documents a rare case of UA in the maxilla of a 3 year old girl.

## Case Report

A 3 year-old girl reported to the outpatient department of Maulana Azad Institute of Dental Sciences, New Delhi, India, with the chief complaint of swelling over left side of jaw since one and a half months. Extraorally, a diffuse swelling of about 2-3 cms in size was noted on the left maxilla. The lesion extended anteriorly up to the ala of the nose and posteriorly up to 2-3 cm in front of tragus. The superio-inferior extent of the swelling was from the floor of the orbit to the ala tragus line. Buccal and lingual expansion was seen intraorally. The swelling was bony hard and non tender on palpation. All primary teeth were present.

**Figure 1:**
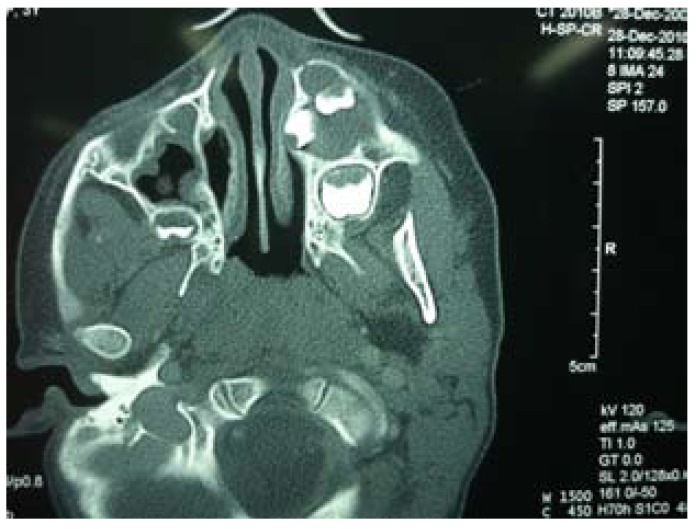
CECT showing corticated cystic lesion involving forming premolars.

Panoramic and occlusal radiographs revealed a well defined radiolucency adjacent to the upper left deciduous canine. Axial CT sections showed an expansile, well corticated cystic lesion involving partially formed maxi-llary left premolars (Fig. 1).

With these findings, a provisional diagnosis of dentigerous cyst was made and the lesion was enucleated under local anaesthesia.

**Figure 2:**
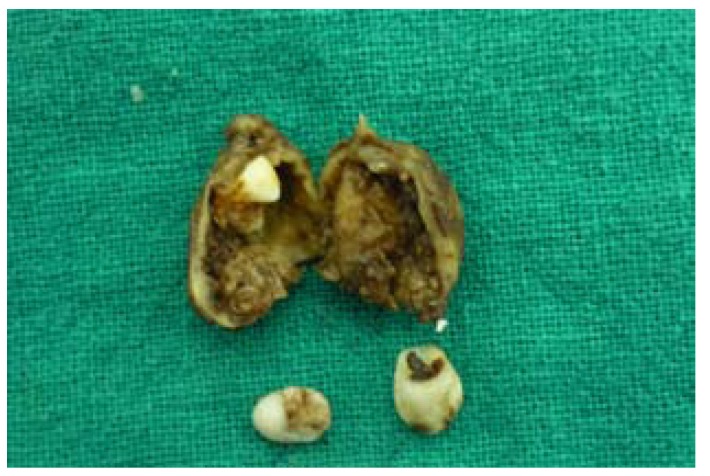
Gross specimen with embedded premolar and multiple mural nodules in cystic sac along with involved canine and premolar.

The gross specimen received was a cystic sac measuring 3.5x3x2.5 cms along with partially formed canine and first premolar. The cystic cavity showed forming 2nd pre molar embedded within the wall of the cyst. Multiple mural nodules were noted in the opened cystic sac (Fig. 2).

**Figure 3:**
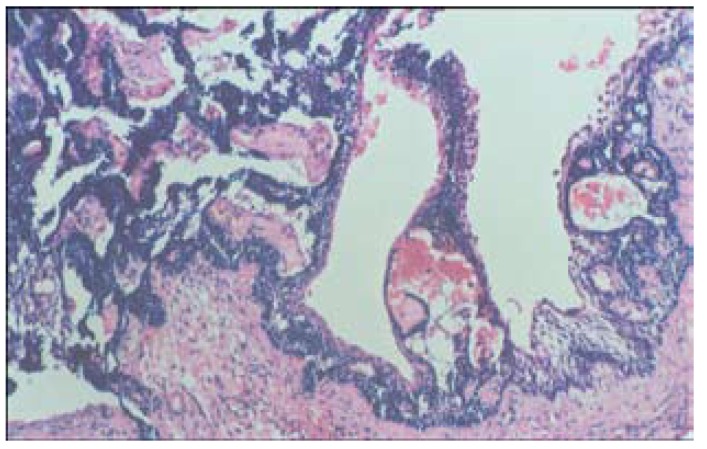
Photomicrograph with characteristic ameloblastomatous lining epithelium proliferating luminally. H&E X10.

Histopathological examination revealed a cystic lining with preameloblast like tall columnar cells in the basal layer. The superficial layers showed loosely arranged oedematous cells resembling stellate reticulum. Luminal and intraluminal proliferation of the odontogenic epithelium in plexiform pattern was observed. Increased vascularity was noted in supportive fibrocollagenous stroma (Fig. 3). Based on these features, a diagnosis of Unicystic Ameloblastoma (Type 1.2) was made. Currently, the patient is under follow up for 15 months and no recurrence has been noted till date.

## Discussion

Conventional ameloblastoma may occur in all age groups with a peak incidence in 3rd & 4th decades of life (7). However, ameloblastoma in younger individuals is thought to be a rare entity, comprising approximately 10% -15% of all reported cases (8). UA is a variant of ameloblastoma that was first described by Robinson and Martinez in 1977 (9).

UA is usually seen in younger patients as compared to solid ameloblastomas, with most tumours diagnosed during the second decade (8). Although there are reports of UA occurring during the first decade of life, the number is limited during the first decade. A thorough search of available literature revealed that the present case of UA in a 3 year old girl is probably the youngest reported patient of UA in the English literature. Mandibular angle and ramus is the favoured site of UA but in the present case maxilla was involved.

Clinically, UA presents as a painless swelling of unknown or relatively short duration (8,10) as was observed in the reported case.

Radiographically, the tumour presents as a unilocular radiolucency in case of dentigerous (tooth associated) variant and rarely as multilocular radiolucency in case of non – dentigerous variant. It is often associated with an unerupted tooth and ranks next to dentigerous cyst as the most frequently occurring pathological pericoronal radiolucency.

Leider et al proposed three pathogenic mechanisms of evolution of UA (11):

1) Reduced enamel epithelium associated with a developing tooth undergoes ameloblastic transformation with subsequent cystic development.

2) Ameloblastomas arise in dentigerous cyst or other types of odontogenic cysts in which the neoplastic ameloblastic epithelium is preceded temporarily by non-neoplastic stratified squamous epithelial lining.

3) Solid ameloblastoma undergoes cystic degeneration of ameloblastic islands with subsequent fusion of multiple microcysts and develops into a unicystic lesion.

Since the present case was associated with a developing permanent teeth, the reduced enamel epithelium attached to them may have undergone ameloblastic transformation lending support to the first hypothesis. However, there is not a solid proof of this origin, as there are several UA that are not related to unerupted teeth and therefore the diverse hypothesis should be viewed with caution.

Histopathologically, UA was classified by Ackerman as (12) :

Type 1 - unilocular, unicystic lesion lined by epithelium.

Type 2 - plexiform variety.

Type 3 - invasive islands of ameloblastomatous epithelium.

Type 3a- islands not connected to cyst lining

Type 3b-islands connected to cyst lining.

Another histopathological grouping by Philipsen and Reichart (13) has also been discussed ( Table 1).

**Table 1:**
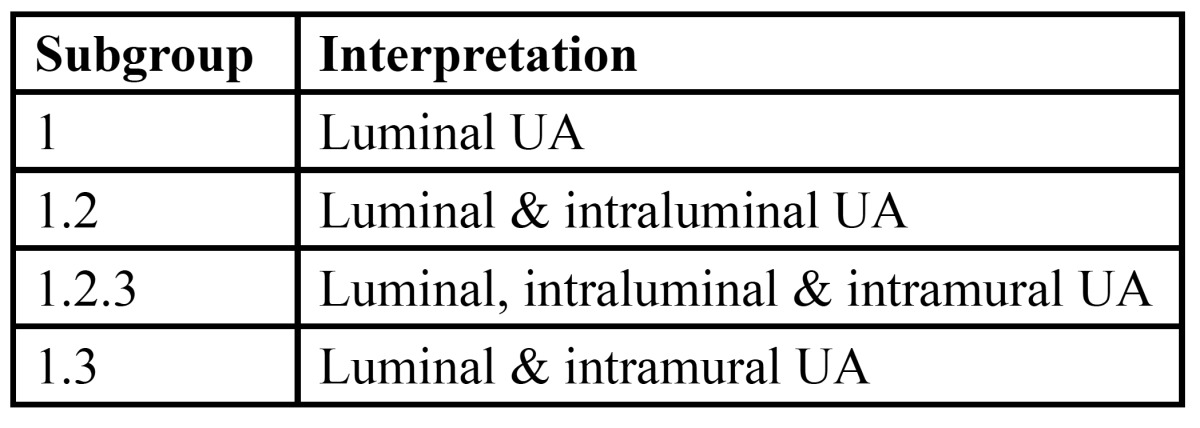
Histopathological grouping by Philipsen and Reichart.

Following this, the published present case falls under 1.2 subgroup.

Unicystic ameloblastoma is generally considered to be a less aggressive tumor compared to the solid variant. However, UA showing intraluminal and intramural proliferation histologically should be considered an aggres-sive lesion and treated in a manner similar to a solid ameloblastoma (14). The occurrence of unilocular radiolucency associated with impacted tooth may lead to an erroneous presumptive diagnosis of a dentigerous cyst by the clinician and result in under treatment. Hence, the importance of pre operative diagnosis by means of an incisional biopsy is reiterated. On the other hand, it may be argued that an incisional biopsy consists of only small fragments and it may be difficult to ascertain the true nature of the lesion even by trained pathologists.

Also, the management of ameloblastoma in a young patient becomes challenging due to concerns about facial growth (7). Therefore, a radically treated ameloblastoma must be followed by an immediate bone graft to pre-vent an obvious facial deformity (14).
